# Electrospun Jets Number and Nanofiber Morphology Effected by Voltage Value: Numerical Simulation and Experimental Verification

**DOI:** 10.1186/s11671-019-3148-y

**Published:** 2019-09-11

**Authors:** Zhi Liu, Kaiyi Ju, Zongqian Wang, Wei Li, Huizhen Ke, Jihuan He

**Affiliations:** 1 Key Laboratory of Textile Fabrics, School of Textile and Garment, Anhui Polytechnic University, No. 8, Beijing Mid-Road, Wuhu, 241000 China; 2 Fujian Key Laboratory of Novel Functional Textile Fibers and Materials, Minjiang University, No. 1, Wenxian-Road, Fuzhou, 350108 China; 3 National Engineering Laboratory for Modern Silk, School of Textile and Clothing Engineering, Soochow University, No. 199, Ren-Ai Road, Suzhou, 215123 China

**Keywords:** Diameter distribution, Electrospinning, Separation, Superfine nanofiber, Voltage

## Abstract

Electrical voltage has a crucial effect on the nanofiber morphology as well as the jet number in the electrospinning process, while few literatures were found to explain the deep mechanism. Herein, the electrical field distribution around the spinning electrode was studied by the numerical simulation firstly. The results show that the electrical field concentrates on the tip of a protruding droplet under relatively low voltage, while subsequently turns to the edge of needle tip when the protruding droplet disappears under high voltage. The experimental results are well consistent with the numerically simulated results, that is, only one jet forms at low voltage (below 20 kV for PVDF-HFP and PVA nanofiber), but more than one jet forms under high voltage (two jets for PVDF-HFP nanofiber, four jets for PVA nanofiber). These more jets lead to (1) higher fiber diameter resulting from actually weaker electrical field for each jet and (2) wide distribution of fiber diameters due to unstable spinning process (changeable jet number/site/height) under high voltage. The results will benefit the nanofiber preparation and application in traditional single-needle electrospinning and other electrospinning methods.

## Introduction

Due to many superior merits such as high surface area, controllable fiber diameter and membrane thickness, and connected pore structure, nanofibers receive intensive studies and have been applied in many areas [1]. As one of the simplest preparation methods of nanofiber, electrospinning technique has drawn numerous attentions not only in academic researches but also in practical industrialization [2, 3].

In view of practical engineering applications, the nanofiber diameter and diameter distribution are the two key parameters. On the one hand, majority of the application areas prefer smaller fiber diameter such as air filtration, because smaller fiber diameter means not only higher surface area which makes the nanofibrous membrane possessing larger pollutants adsorption capacity but also smaller pore size endowing the nanofibrous membrane higher pollutants repelling ability [4, 5]. Many methods have been developed to pursue finer nanofiber. For example, adding ionic/inorganic salt can be an effective way because the salt can increase the spinning fluid conductivity [6, 7]. Wang et al. reported that increasing the sheath fluid flow rate can reduce the resulting nanofiber diameter in the coaxial spinning process [8]. Hai et al. developed a detachable concentric spinneret that can hold the energy on working fluid by the outer polymeric tube, which benefits preparing much finer core-shell nanofiber [9]. On the other hand, narrow diameter distribution results in better control of pore size in the nanofibrous membrane construction, which is crucial in separation areas especially in water filtration [10, 11].

In the spinning process, many parameters from the device and precursor solutions are involved in the nanofiber diameter and diameter distribution. First, the shape of the spinning electrode plays a significant role in determining the electrical field distribution and, as a result, has an important influence on the spinning process and nanofiber morphology [12, 13]; second, the precursor properties such as the concentration, surface tension, and viscosity [14, 15]; third, spinning parameters such as voltage, collector distance, and even the collector shape [16, 17]; fourth, ambient conditions such as the humidity and the temperature [18]. Among them, the voltage value has much crucial effect on nanofiber diameter and diameter distribution, though those parameters synergistically affect the spinning process and nanofiber morphology [19].

Theoretically, the nanofiber diameter decreases with the increase of voltage value where electrical field force is strengthened [20]. Therefore, increasing voltage value can be a feasible route to achieve superfine nanofiber [21]. Hasanzadeh et al. [22] reduced the polyacrylonitrile nanofiber diameter from 212 to 184 nm using the applied voltage from 14 to 22 kV. Ranjbar-Mohammad et al. [23] fabricated gum tragacanth/poly (vinyl alcohol) composite nanofiber and achieved the decrease of fiber diameter from 153 to 98 nm by changing the voltage from 10 to 20 kV. However, interestingly, for traditional single-needle electrospinning (TNE), there are two phenomena at high voltage value in spinning process: (1) higher fiber diameter. It is well known that the nanofiber diameter decreases with the increase of voltage value at first, while increases at high voltage value [24]; (2) wide fiber diameter distribution. Wide fiber diameter distribution is achieved at high voltage value in the TNE spinning process [25]. That is to say that higher voltage value is unwelcome in TNE spinning process. As a result, it is a hard task to obtain nanofiber with smaller diameter and narrow diameter distribution due to the limited voltage value in TNE spinning process.

Therefore, the relevant mechanism discussion is greatly desired to reveal the phenomenon and benefits of the nanofiber preparation. However, little literatures report the mechanism of the phenomenon that TNE method prepares nanofiber with a higher diameter and wider diameter distribution under high voltage value. Many previous researches applied the numerical simulation method by Maxwell program to intuitively evaluate the electrical field distribution and intensity of electrospinning apparatus [26–28]. In the present study, we research the mechanism in a special view and aim to (1) numerical simulation of electrical field distribution around the spinning electrode in TNE spinning process with voltage supply change, (2) experimental verification of numerical simulation results and voltage value on the spinning process and nanofiber morphology, and (3) spinning process conclusion with the increase of voltage value and mechanism discussion of abnormal nanofiber morphology under high voltage value.

## Methods

### Materials

Poly (vinylidene fluoride-co-hexafluoropropylene) (PVDF-HFP, *Mw* = 400,000) was purchased from Aladdin Industrial Corporation, Shanghai, China. Polyvinyl alcohol (PVA), *N*,*N*-dimethyl formamide (DMF), and acetone were supplied by Sinopharm Chemical Reagent Co., Ltd. (Suzhou, China). All reagents were analytical grade and were used as received without further treatment.

### Preparation of PVA Nanofiber Under Different Voltage Value

PVDF-HFP (11 wt%) was dissolved in a binary solvent of DMF/acetone with the weight ratio of 1:1 at room temperature for 4 h. In spinning experiment, the voltage values of 6, 10, 15, 20, 25, and 30 kV were applied at the tip of a syringe needle (0.8 mm in internal diameter). The collector distance is 15 cm. A constant volume flow rate of 1.0 ml/h was maintained using a syringe pump. The temperature and relative humidity (RH) used in the spinning process were 25 ± 2 °C and 55 ± 3%, respectively, and kept constant.

### Preparation of PVA Nanofiber Under Different Voltage Value

PVA (12 wt%) was dissolved in deionized water at 95 °C for 2 h. The sodium dodecylbenzenesulfonate (0.01%) was added into the solution to decrease the solution surface tension. In spinning experiment, the voltage values of 7, 10, 15, 20, 25, and 30 kV were applied at the tip of a syringe needle (0.8 mm in internal diameter). The collector distance is 15 cm. A constant volume flow rate of 0.8 ml/h was maintained using a syringe pump. The temperature and RH used in the spinning process were 25 ± 2 °C and 55 ± 3%, respectively, and kept constant.

### Characterization

The morphology of electrospun nanofibrous membranes was observed using a scanning electron microscope (Hitachi S-4800, Tokyo, Japan) at 20 °C, 60 RH. Samples were sputter-coated with gold layer prior to imaging. The samples were cut up into 2 × 4 mm^2^ and photographed at accelerating voltage of 5 kV and electricity of 10 mA. The diameters of electrospun fibers were calculated by measuring at least 100 fibers at random using *ImageJ* program. The optical images were photographed by a camera (SONY, ILCE-6400L). In the photographing process, a black plank was placed at the back and a torch was placed opposite from the camera lens, which can photograph the spinning process with a high quality.

In the numerical simulation process, the electric field around the spinning electrode was calculated by using Maxwell 2D (ANSOFT Corporation). The simulation parameters are the outer and inner diameter of the needle are 1.2 mm and 0.8 mm, respectively; the length of three protruding droplet lengths are 1.3 mm, 0.88 mm, and 0 mm, respectively; and the collector distance is 15 cm. The Maxwell program utilizes finite element methods and adaptive meshing to achieve a converged solution. In the simulation process, the calculation finished at Energy Error and Delta Energy are less than 1%. The conductivity of model polymeric solution in simulation process is 1.6 μs/cm.

## Results and Discussions

### Schematic Diagram of Jet Evolution and Numerical Simulation of the Electrical Field Around the Electrode with Voltage Value Change

**Fig. 1:**
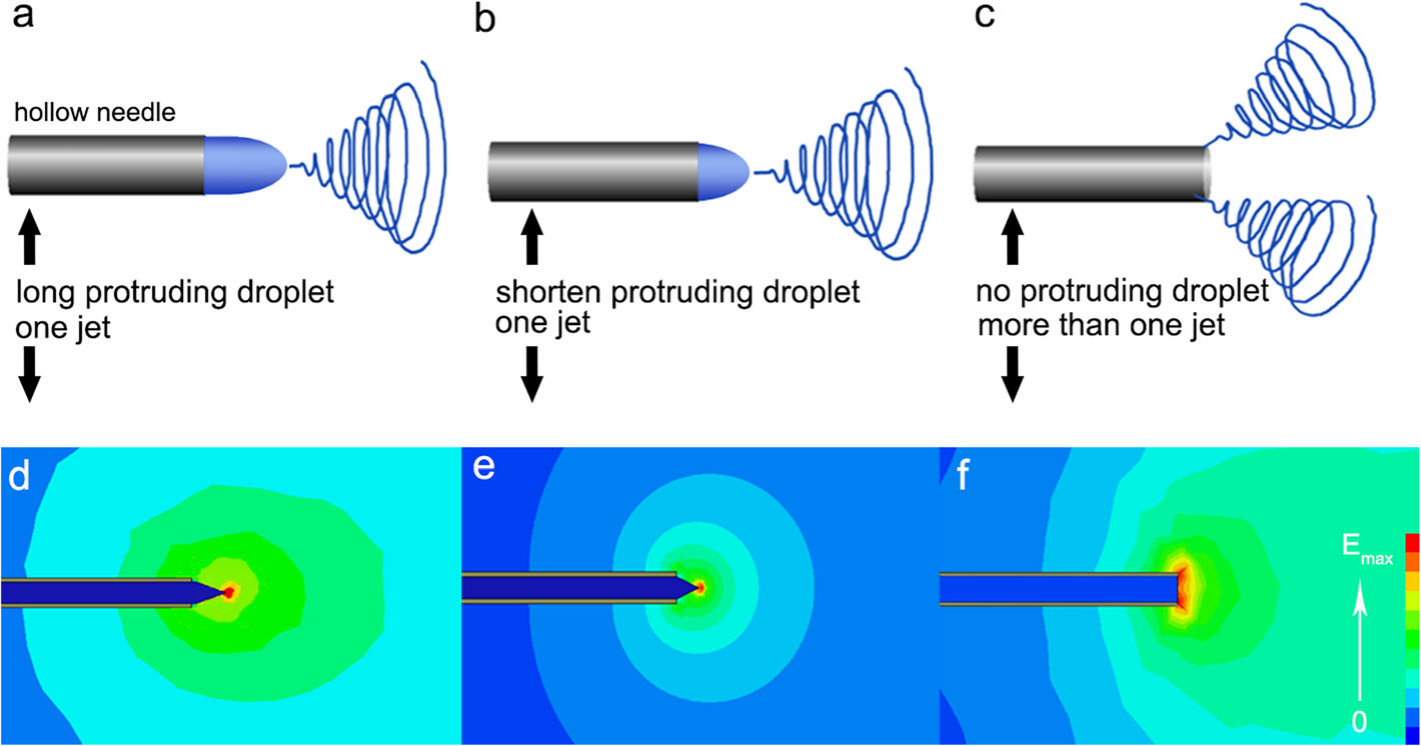
**a**–**f** The schematic diagram of the jet evolution and the electrical field distribution at three protruding droplet lengths (long protruding droplet, short protruding droplet, no protruding droplet)

In the spinning process, various parameters affect the resulting nanofiber/particles diameters, as reported by Huang et al. [29]; the fluid jet length and the fluid jet angle can be helpful to predicting the diameters of the resulting nanofiber/particles. In the TNE spinning process, the protruding droplet length will decrease with the increase of voltage value (Fig. 1a–c) [30]. Three protruding droplet lengths: long protruding droplet, short protruding droplet, and no protruding droplet are simulated, respectively (Fig. 1). As shown in Fig. 1a, at low voltage value, the polymer solution forms a long protruding droplet on needle tip due to weak electrical force. In this situation, the electrical field concentrates on the tip of protruding droplet (Fig. 1d). Therefore, we can speculate that there will be only one jet generating from the protruding droplet tip at this circumstance (Fig. 1a). With the increase of voltage value, the protruding droplet length decreases due to the stronger electrical field force (Fig. 1b), which is in accordance with the previous study that the height of Taylor cone gradually decreased as the applied voltage increased from 13 to 16 kV [31]. And the electrical field concentrates on the tip of protruding droplet as well (Fig. 1e), resulting in one polymeric jet still (Fig. 1b). However, with the voltage value increase to a critical value, the protruding droplet disappears (Fig. 1c), and the strongest electrical field turns to the tube edge of the needle tip (Fig. 1f). At this situation, more than one jet will be formed along the tube edge of the needle tip (Fig. 1c).

**Fig. 2:**
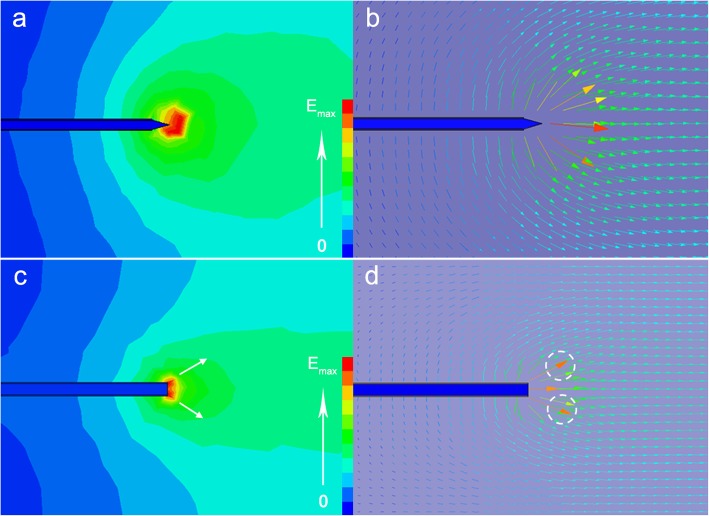
**a** The electrical field distribution and **b** the velocity vector diagram around the needle tip (protruding droplet exists); **c** the electrical field distribution and **d** the velocity vector diagram around the needle tip (no protruding droplet exists)

The velocity vector diagram can be an effectively indicator for the polymeric jet number and jet direction [32]. Therefore, the velocity vector plot around the needle tip was simulated in Fig. 2b, d, where the arrows denote the velocity direction and the arrow length and color represent the value. The longest arrow with deep red color is the site that the polymeric jet generates from. As illustrated in Fig. 2b, the red color and the longest arrow are in the ahead of the solution tip where the only one jet forms, which is in accordance with the electrical field distribution diagram that electrical field intensives on the protruding droplet tip (Fig. 2a). Differently, the electrical field intensives on the tube edge of needle tip when there is no protruding droplet appearance (Fig. 2c). Meanwhile, the relatively longest and red color arrows trigger from the needle tube edge (Fig. 2d). As a result, more than one jet generates from the tube edge of the needle tip (Fig. 1c).

**Fig. 3:**
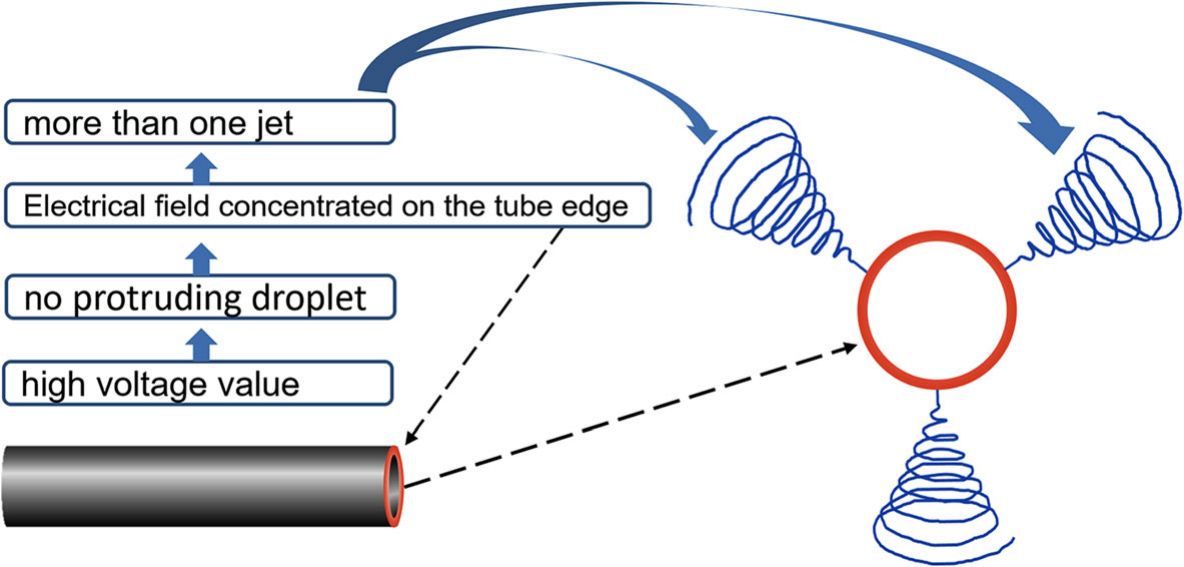
Schematic diagram of the electrical field distribution and jet number with no protruding droplet under high voltage value

Specially, under high voltage value, the protruding droplet will disappear, then the electrical field concentrates on the tube edge; subsequently, it forms more than one jet around the needle tip (Fig. 3), which has a great effect on the spinning process and nanofiber morphology. As shown in Fig. 3, it is speculated that the more jet numbers favor to two results: (1) the weaker electrical field for each jet—in spite of under high voltage value, the increased jets share the limited electrical field, resulting in weakened electrical field for each jet actually, which contributes to prepare the nanofiber with big fiber diameter—and (2) unstable spinning process. In this situation, both the different electrical field intensity of each jet and changeable jet number, jet site conduces to unstable spinning process. As a consequence, this unstable spinning process favors to a worse fiber uniformity with wide nanofiber diameter distribution and even bad nanofiber morphology, which shows bad effect on the membrane property such as the membrane porosity and membrane pore size distribution [33], subsequently, poor performance in some practical applications.

### Experimental Verification by Electrospun PVDF-HFP Nanofiber

**Fig. 4:**
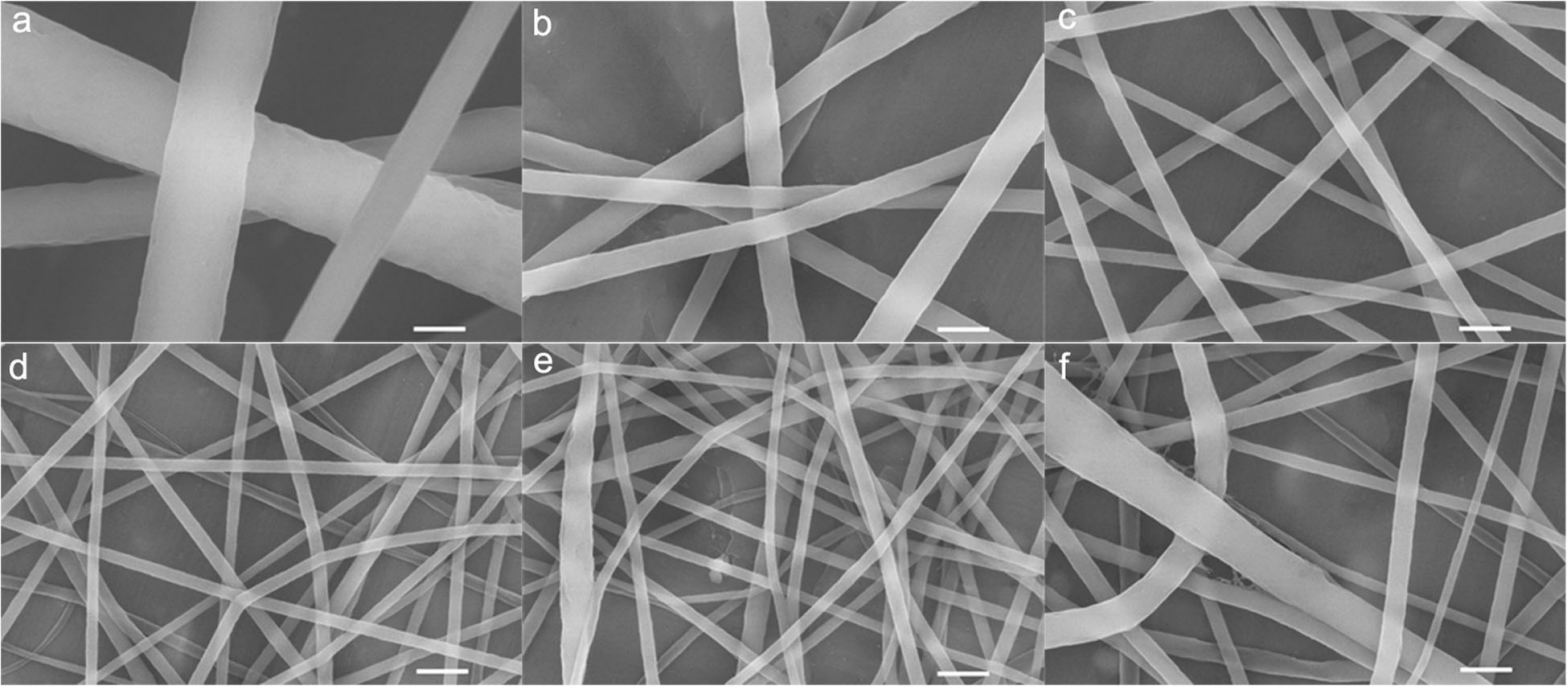
The morphology of PVDF-HFP nanofiber under different voltage value **a** 6 kV, **b** 10 kV, **c** 15 kV, **d** 20 kV, **e** 25 kV, and **f** 30 kV (the scale bar is 600 nm)

To confirm the effect of voltage value change on the nanofiber morphology, the PVDF-HFP nanofibers were fabricated under different voltage value. As illustrated in Fig. 4, the PVDF-HFP nanofibers show smooth surface at all voltage value. Meanwhile, with the increase of voltage value, the PVDF-HFP nanofiber diameter decreases at first (1004.3 ± 184.7 nm at 6 kV, 387.4 ± 46.6 nm at 10 kV, 239.5 ± 20.4 nm at 15 kV, 149.2 ± 9.5 nm at 20 kV) (Table 1) (Fig. 4a–d), which results from the increase of electrical field force induced by the increased voltage value. However, the fiber diameter increases gradually at voltage 25 kV (194.2 ± 47.9 nm) (Table 1, Fig. 4e) and 30 kV (247.9 ± 59.6 nm) (Table 1, Fig. 4f). Moreover, the nanofiber shows narrow diameter distribution firstly, while presents bad dimeter distribution at voltage 25 kV (Fig. 4e) and worse at voltage 30 kV (Fig. 4f).

**Table 1:** The average diameter of PVDF-HFP nanofiber under various voltage value

Voltage (kV)	6	10	15	20	25	30
Diameter (nm)	1004.3 ± 184.7	387.4 ± 46.6	239.5 ± 20.4	149.2 ± 9.5	194.2 ± 47.9	247.9 ± 59.6

**Fig. 5:**
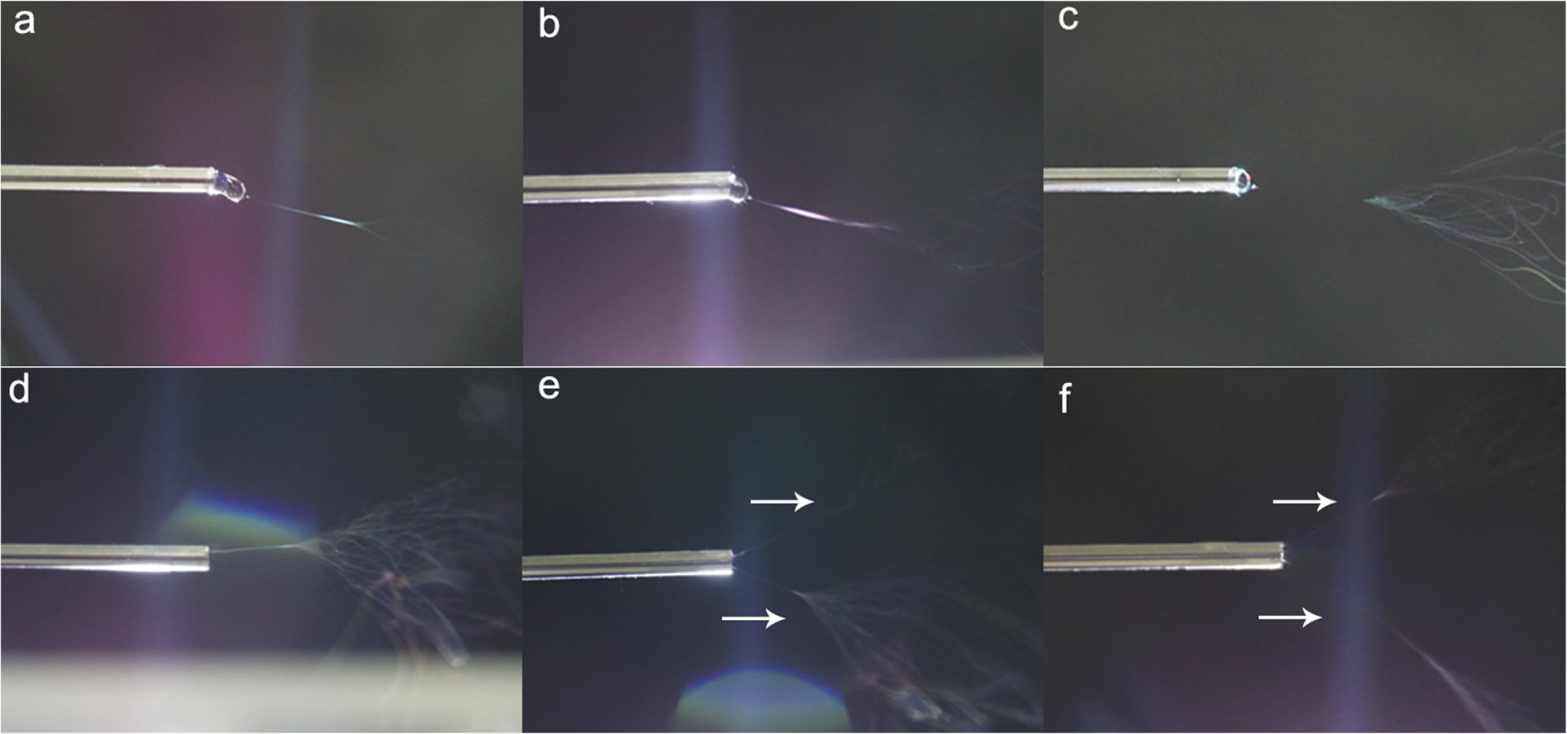
The optical images of jet evolution in spinning process under different voltage value **a** 6 kV, **b** 10 kV, **c** 15 kV, **d** 20 kV, **e** 25 kV, and **f** 30 kV (the inner diameter of spinneret is 0.8 mm, outer diameter of spinneret is 1.2 mm)

To confirm the effect of voltage value change on the jet number in spinning, the jet evolution process under different voltage value is shown in Fig. 5. It can be seen that the protruding droplet length decreases with the increase of voltage value from 6 to 20 kV (Fig. 5a–d). What is more, only one jet initiates at the voltage value lower than 20 kV, which agrees with the numerical simulation results that the electrical field concentrating on the droplet tip produces one jet before the protruding droplet disappearance. However, with the increase of voltage value, the protruding droplet disappears and two jets form at the needle tip (Fig. 5e, f). These results further confirm the numerical simulation results that it forms more than one jet due to the stronger electrical field turning to tube edge of the needle tip under high voltage value.

**Fig. 6:**
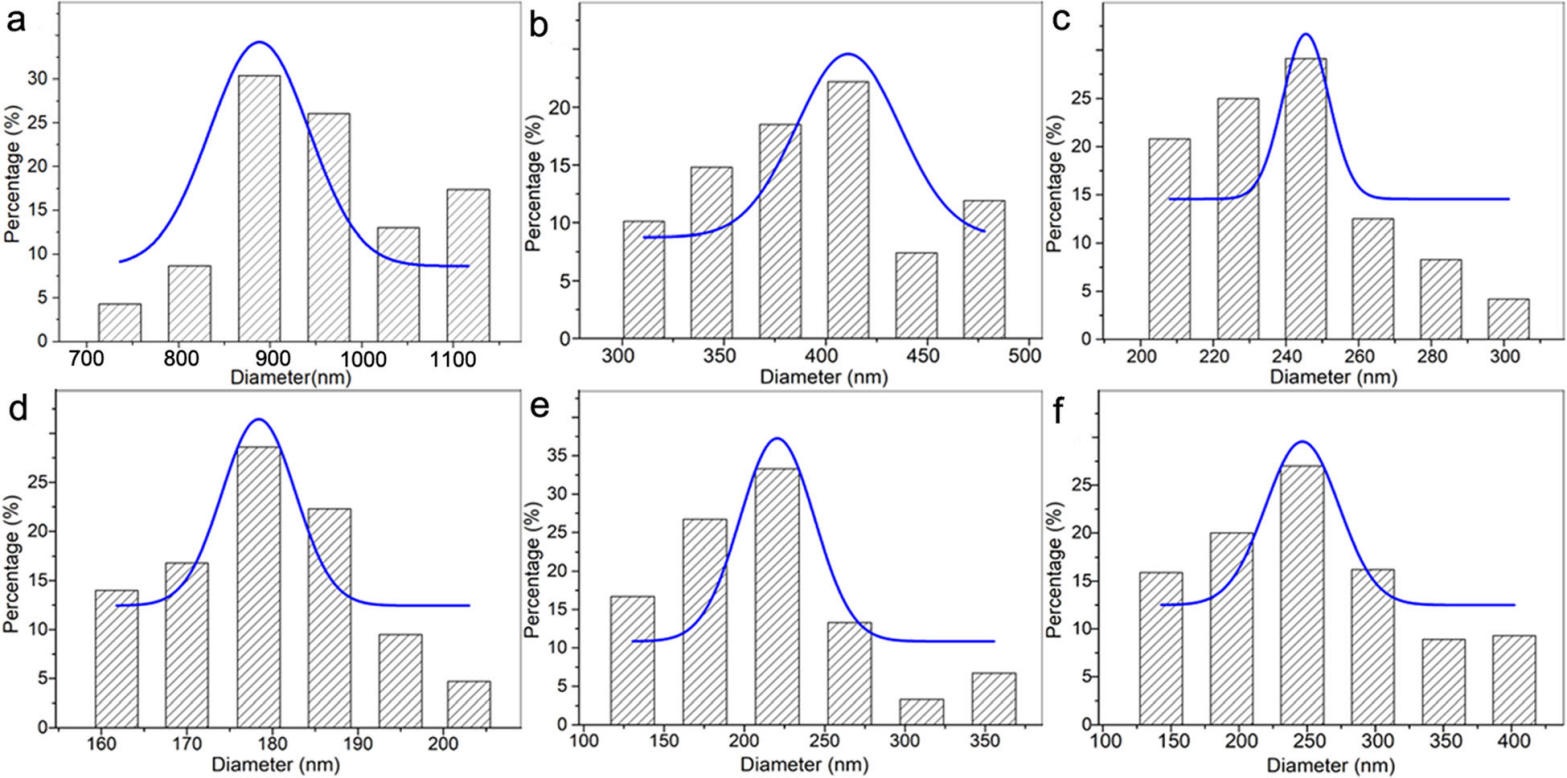
The diameter distribution of PVDF-HFP nanofiber under different voltage value **a** 6 kV, **b** 10 kV, **c** 15 kV, **d** 20 kV, **e** 25 kV, and **f** 30 kV

The diameter distribution is a crucial indicator for practical application, especially in separation areas such as precise water filtration which needs a narrow pore distribution effected by the diameter distribution. As shown in Fig. 6a, the fiber diameter is 1004.3 ± 184.7 nm with diameter distribution from 495.1 to 1347.9 nm at voltage value 6 kV. For voltage 10 kV and 15 kV, the fiber diameter is 387.4 ± 46.6 nm and 239.5 ± 20.4 nm, respectively, with a narrow diameter distribution (Fig. 6b, c). At the voltage of 20 kV, the fiber diameter is 149.2 ± 9.5 nm with a considerably narrow diameter distribution from 157.6 to 207.5 nm (Fig. 6d). At the voltage value 25 kV, the fiber diameter is 194.2 ± 47.9 nm with a wide diameter distribution from 108.7 to 377.8 nm (Fig. 6e). The fiber diameter increases to 247.9 ± 59.6 nm with much wider diameter distribution from 117.2 to 428.3 nm at the voltage value 30 kV (Fig. 6f). It can be seen that the PVDF-HFP nanofiber with relatively narrow diameter distribution when the voltage values are less than 20 kV. Beyond the voltage of 20 kV, the PVDF-HFP nanofiber shows worse uniformity with increased average fiber diameter. These results further demonstrate that the fiber diameter decreases firstly followed by increase with the increase of voltage value. Furthermore, it shows wide diameter distribution at high voltage value, which is in well accordance with the numerical simulation results and previous studies [34].

### Experimental Verification by Electrospun PVA Nanofiber

**Fig. 7:**
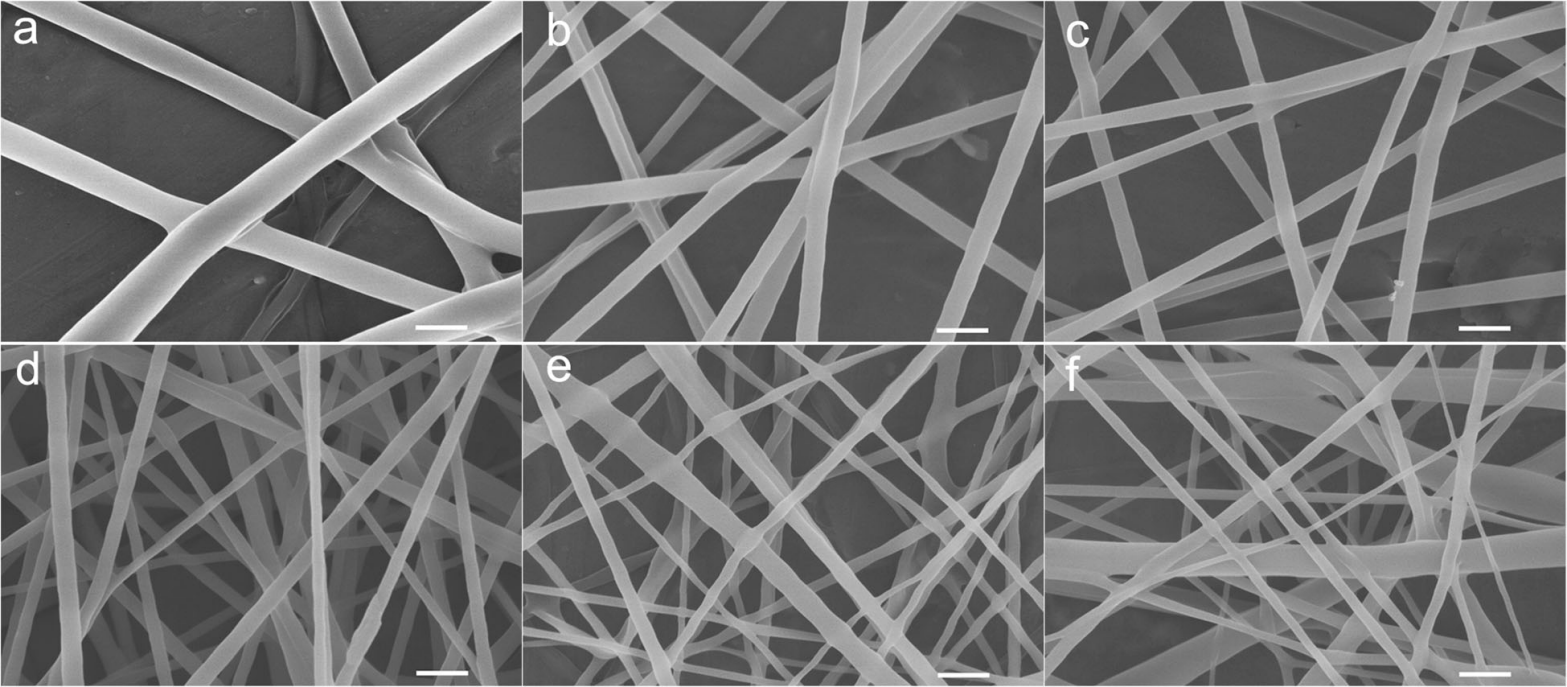
The morphology of PVA nanofiber under different voltage value **a** 7 kV, **b** 10 kV, **c** 15 kV, **d** 20 kV, **e** 25 kV, and **f** 30 kV (the scale bar is 600 nm)

**Fig. 8:**
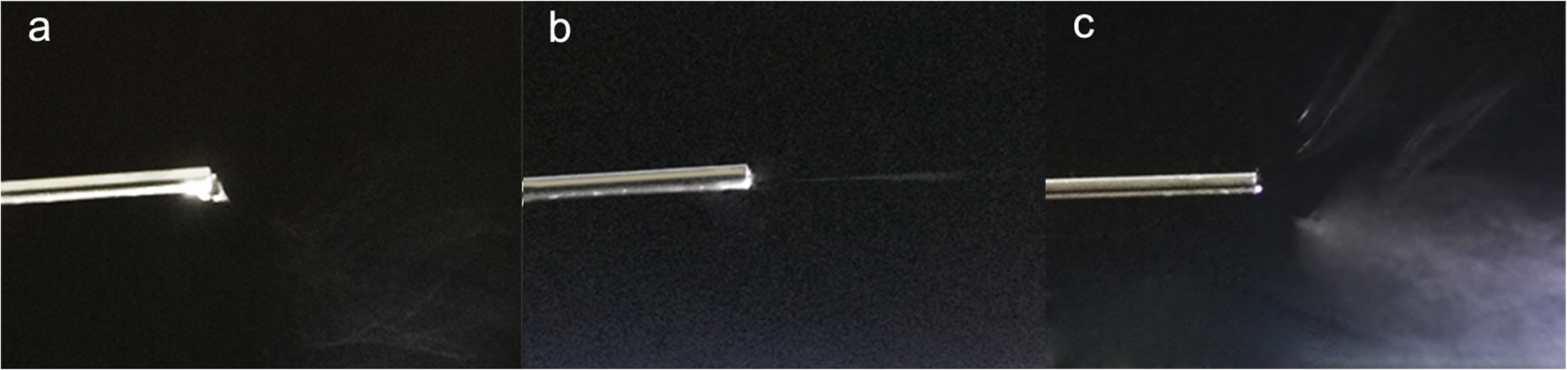
The optical images of jet number in spinning process under voltage value **a** 15 kV, **b** 20 kV, and **c** 30 kV (the inner diameter of spinneret is 0.8 mm, outer diameter of spinneret is 1.2 mm)

**Fig. 9:**
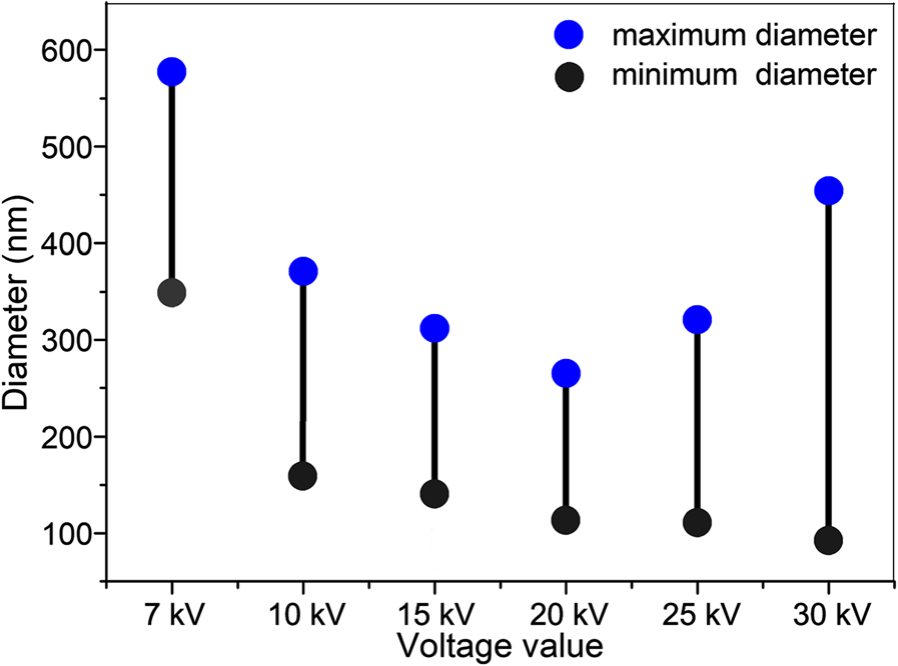
The diameter distribution of PVA nanofiber under different voltage value

To further confirm the effect of voltage supply change on the nanofiber morphology and jet number evolution in spinning, the PVA nanofibers were fabricated under different voltage value. As shown in Fig. 7, with the increase of voltage value, the PVA nanofiber diameter decreased at first (voltage value less than 20 kV), accompanied by increase gradually at voltage 25 kV (186.7 ± 43.4 nm) and 30 kV (213.6 ± 64.9 nm). These results are in well keeping with the PVDF-HFP nanofibers. The jet evaluation with voltage value (15, 20, and 30 kV) is shown in Fig. 8. It is can be seen that the protruding droplet length decreases and generates only one jet from the protruding droplet tip at voltage value of 15 and 20 kV (Fig. 8a, b). However, at voltage value 30 of kV, more than one jet formed at the needle tip (Fig. 8c). The increased jets lead to two results: (1) higher average diameter which is confirmed by the diameter change (Table 2, Fig. 9) and (2) worse diameter distribution clearly shown in Fig. 9 that the gap between the minimum and maximum diameter shows a decrease tendency (228 nm at 7 kV, 212 nm at 10 kV, 169 nm at 15 kV, 149 nm at 20 kV,) but a dramatical increase to 202 nm at 25 kV and 361 nm at 30 kV.

**Table 2:** The average diameter of PVA nanofiber under various voltage value

Voltage (kV)	7	10	15	20	25	30
Diameter (nm)	442.7 ± 59.8	272.8 ± 35.7	232.9 ± 29.0	159.3 ± 23.6	186.7 ± 43.4	213.6 ± 64.9

### Spinning Process Conclusion with the Voltage Value Increase and Mechanism Discussion of Jet Evolution Affecting the Spinning Process and Nanofiber Morphology

**Fig. 10:**
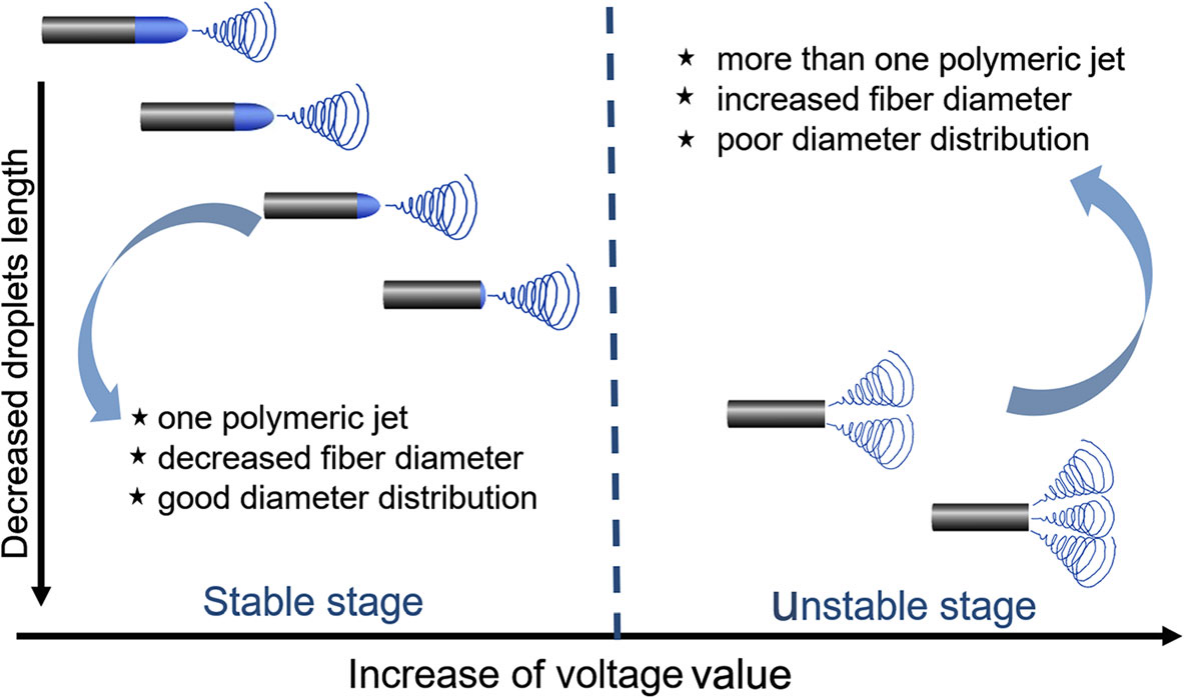
The schematic diagram of spinning process and jet evolution with the increase of voltage value

Based on the numerical simulation and experimental verification results, the spinning process with the voltage value increase and the mechanism of jet evolution affecting the nanofiber spinning process and morphology are tentatively concluded as follows:

As shown in Fig. 10, the protruding droplet length decreases firstly and disappears gradually with the increase of voltage value. Meanwhile, the electrical field intensifies on protruding droplet tip firstly and then turns to the tube edge of the needle tip. These two phenomena lead to only one jet forms at the protruding droplet existing before the protruding droplet disappearance and more than one jet forms after the protruding droplet disappearance (Fig. 10).

Therefore, the spinning process can be reasonably separated by two stages, before and after protruding droplet disappearance or stable and unstable stage (Fig. 10). Before the protruding droplet disappearance (stable stage), the fiber diameter decreases with the voltage value increase and shows relatively good diameter distribution. After the protruding droplet disappearance (unstable stage), (1) the fiber diameter increases oppositely due to the weaker electrical field for each jet which is actually due to the increased jet number and (2) there was worse fiber diameter distribution contributed by the unstable spinning process (changeable jet number, jet sit, and different electrical field intensity for each jet). In view of the discussions above, the critical value before protruding droplet disappearance is the best voltage value to fabricate nanofiber with finer fiber diameter and good fiber diameter distribution (Fig. 10).

## Conclusions

The numerical simulation and experimental verification results show that only one jet forms at the protruding droplet exists and more than one jet produces after the protruding droplet disappearance, which is contributed by the electrical field concentrating on droplet tip firstly and then turning to the tube edge of needle tip with the increase of voltage value. The increased jet not only weakens the electrical field for each jet (resulting in high fiber diameter), but also makes an unstable spinning process (leading to wide diameter distribution). The results ingeniously reveal the mechanism of nanofiber morphology change at high voltage value in TNE spinning process, which presents a unique view to better know the TNE spinning process and benefits the nanofiber preparation and application in many areas especially in separation and filtration.

## Data Availability

The data in the present study are available from the corresponding authors based on a reasonable request.

## Abbreviations

DMF: *N*,*N*-Dimethyl formamide
PVA: Polyvinyl alcohol
PVDF-HFP: Poly (vinylidene fluoride-co-hexafluoropropylene)
RH: Relatively humidity
TNE: Traditional single-needle electrospinning
